# Supplementary material for: MetaRibo-Seq measures translation in microbiomes
Source: Nat Commun. 2020 Jun 29;11:3268. doi: 10.1038/s41467-020-17081-z (PMC7324362; doi:10.1038/s41467-020-17081-z)
Supplement: Supplementary file 10 — Supplementary Data 7 [file 41467_2020_17081_MOESM10_ESM.zip › File2/Confidence_High_Taxonomy/410114_out.krona.html]

Javascript must be enabled to view this page.

members
magnitude
magnitudeUnassigned
count
unassigned
taxon
rank

410114\_out

6

6
2
superkingdom

6
976
phylum

200643
class
6

6
171549
order

family
815
6

816
genus
6

6

SRS013940\_contig\_number\_21200SRS019286\_contig\_number\_contig-100\_1665.36701SRS056273\_contig\_number\_14245SRS075078\_contig\_number\_19120SRS104636\_contig\_number\_554SRS147139\_contig\_number\_678
1263049
species
